# Comparative Assessment of Cellular Responses to Microscale Silica Morphologies in Human Gastrointestinal Cells: Insights for Occupational Health

**DOI:** 10.3390/ijerph21101376

**Published:** 2024-10-18

**Authors:** Mohammad Z. Yamin, James Y. Liu, Christie M. Sayes

**Affiliations:** Department of Environmental Science, Baylor University, One Bear Place #97266, Waco, TX 76798-7266, USA; mohammad_sheikhyami1@baylor.edu (M.Z.Y.); james_liu@baylor.edu (J.Y.L.)

**Keywords:** Caco-2, mitochondrial activity, pyrogenic, diatomaceous, crystalline, exposure

## Abstract

Silicon dioxide (SiO_2_), commonly known as silica, is a naturally occurring mineral extracted from the Earth’s crust. It is widely used in commercial products such as food, medicine, and dental ceramics. There are few studies on the health effects of pyrogenic and colloidal silica after ingestion. No research has compared the impact of microscale morphologies on mitochondrial activity in colon cells after acute exposure. The results show that crystalline and amorphous silica had a concentration-independent effect on cells, with an initial increase in mitochondrial activity followed by a decrease. Vitreous silica did not affect cells. Diatomaceous earth and pyrogenic silica had a concentration-dependent response, causing a reduction in mitochondrial activity as concentration increased. Diatomaceous earth triggered the highest cellular response, with mitochondrial activity ranging from 78.84% ± 12.34 at the highest concentration (1000 ppm) to 62.54% ± 17.43 at the lowest concentration (0.01 ppm) and an average H_2_O_2_ concentration of 1.48 ± 0.15 RLUs. This research advances our understanding of silica’s impact on human gastrointestinal cells, highlighting the need for ongoing exploration. These findings can improve risk mitigation strategies in silica-exposed environments.

## 1. Introduction

Silicon is the second most abundant element in the world [1]. It is divided into two main forms: crystalline and amorphous [2]. Silica is naturally sourced via rock erosion, volcanic eruptions, and biogenic activities [3]. Here, we introduce five silica types: one crystalline (quartz) and four amorphous (colloidal, vitreous, pyrogenic, and diatomaceous earth).

Crystalline silica is a naturally occurring mineral found abundantly in the Earth’s crust [4]. These crystalline structures impart specific properties, including hardness and electrical insulation [5]. Manufacturing processes involve extraction from natural sources such as quartzite or sandstone [6,7]. The physicochemical features allow crystalline silica to be widely used across industries. For instance, in construction, it is a key component in concrete, ceramics, and bricks due to its reinforcement properties [8]. In electronics, chips and semiconductors are manufactured using silica crystals [9]. However, crystalline silica poses health risks due to the silanol group (SiOH) [10], especially in occupational settings where exposure to respirable crystals can lead to lung diseases such as silicosis [11]. Crystalline silica exists in polluted water at concentrations up to 100 ppm [12]. However, while there is a lack of health hazard data after silica ingestion, consuming quartz-contaminated drinking water poses an exposure risk for the general population [3]. It is necessary to delve deeper into its detrimental health impacts when consumed. Research in this area ought to be expanded, largely due to the lack of documented studies in the literature.

Colloidal silica consists of fine, stable, and amorphous particles suspended in a liquid medium. Manufacturing processes typically involve the controlled hydrolysis and condensation of silicate precursors in aqueous solution, forming stable colloidal suspensions at different sizes [13,14]. These particles exhibit unique properties such as high surface area, stability, and transparency [15,16]. Colloidal silica is used in concrete admixtures, mortars, grout repair, and insulating materials due to its ability to enable mixture homogeneity, which reduces water penetration and surface cracking. It also helps increase mechanical strength and thermal insulation, making it ideal for roofs and walls [17,18]. While colloidal silica is not allowed as a food additive [19,20], it is still present in water bodies, posing a threat through ingestion.

Pyrogenic silica, or fumed silica, is produced through the high-temperature hydrolysis of silanes or quartz sand in a flame reactor [2,21]. Fumed silica is pure and reactive, making it especially desirable for alkali-free synthesis due to its minimal sodium content [22]. It exhibits a low bulk density alongside a high specific surface area, typically between 200 m^2^/g and 300 m^2^/g [23]. Nonetheless, its ultra-light and airy nature presents challenges in handling the powder [22]. Pyrogenic silica has various uses, including as a reinforcing filler, a thickening agent, in cosmetics and pharmaceuticals, and as a food additive to prevent clumping [19,24]. By 2026, it is anticipated that the utilization of pyrogenic silica will see a 2.3% increase from its 2021 levels [25]. With studies demonstrating the respirable effects of nanoscale silica, it is essential to investigate how ingested pyrogenic silica impacts cellular health.

Vitreous silica, also known as fused silica, is an amorphous form of silica obtained by melting high-purity silica sand or quartz at high temperatures and then rapidly cooling the melt to form a non-crystalline solid [2,3]. Vitreous silica is widely employed in producing glassware, optical fibers, and specialty ceramics. Its exceptional properties make it suitable for applications requiring high-performance materials, such as lenses, prisms, and photonic crystals [26,27]. Research on the impact of silica on human gut models is limited; the focus should lie on understanding its effects in scenarios of accidental exposure.

Diatomaceous earth (DE), a naturally occurring sedimentary rock, is composed of the fossilized remains of diatoms, which are a significant group of single-celled, hard-shelled algae with silica-based cell walls that have settled at the bottom of rivers, lakes, and oceans over thousands or millions of years [28,29]. Within DE, the silica is mostly amorphous, with individual silica particles having an average diameter of fewer than 50 μm. These particles exhibit high porosity, low thermal conductivity, a high melting point, and chemical inertness [30]. It is mined from deposits and processed to remove impurities and refine particle size [31,32]. DE finds diverse applications across industries. In filtration, it is used as a porous medium for water and beverage purification [33,34]. In agriculture, DE serves as an insecticide and soil amendment, providing natural pest control and improving soil fertility [35]. Additionally, DE is used in some dietary supplements due to its purported detoxifying properties [36].

Silica additives, identified as E551 in food products, are recognized for their lack of acute toxicity responses in intestinal epithelial cells [20]. However, this paper compares the induced cellular health effects across five different silica types, critical for the industrial workplace, such as in manufacturing processes like sandblasting or silica nanoparticle production. While the primary concern with silica exposure is inhalation, particles can be ingested. For instance, workers might inadvertently swallow different amounts of silica dust while working or ingest contaminated water or food in areas where silica dust is present. For example, silica was detected as a contaminant in drinking water at concentrations ranging from 3.3 mg/L to 7.1 mg/L [3,37,38]. It was observed in the form of dissolved silica in groundwater (14 mg/L), surface water (up to 6 mg/L), and deep water (up to 10.8 mg/L) [3,39]. Alternatively, additional research indicated that the average overall silica concentration in certain groundwater sources ranged from 15 mg/L to 65 mg/L, with occasional peaks reaching up to 120 mg/L [40]. Due to the geographic distribution of power plants and manufacturing facilities globally, the levels of silica in source waters can vary widely, ranging from 1 to 60 parts per million (ppm) and even reaching up to 300 ppm in some volcanic areas [41].

While each form of silica offers unique properties and applications, its induced effects on Caco-2 cells underscore potential implications for gut health. Understanding these distinctions is crucial for assessing the safety and suitability of silica-based products across industries and applications. The underlying cellular responses to silica exposure must be elucidated to ensure safe and sustainable material development. The primary aim of this study is to investigate the impact of different microscale silica particle morphologies on cellular responses upon accidental ingestion of contaminated food and water, particularly in occupational settings. This study investigates mitochondrial activity and reactive oxygen species (ROS) production in human gastrointestinal cells by directly exposing Caco-2 cells to silica. By isolating factors such as the mucus layer and intestinal microbiota, we aim to better understand silica’s toxicity while minimizing potential confounding influences. Focusing on the Caco-2 model allows us to understand the specific cellular responses to silica.

## 2. Materials and Methods

### 2.1. Materials and Reagents

Crystalline, colloidal, vitreous, pyrogenic, and diatomaceous earth silica particles were purchased at the highest purity. Table 1 presents the details associated with each type of purchased silica. Dulbecco’s modified eagle medium nutrient mixture F-12 (DMEM/F-12), Dulbecco’s phosphate-buffered saline (PBS), penicillin–streptomycin solution, fetal bovine serum (FBS), 0.25% trypsin/EDTA (ethylenediaminetetraacetic acid) solution, 2-Methyl-1, 4-naphthoquinone, 98% (Menadione), and Triton X-100 were purchased from Thermo Fisher Scientific (Waltham, MA, USA). Human epithelial colorectal adenocarcinoma cells (Caco-2, HTB-37) were purchased from the American Type Culture Collection (ATCC) (Manassas, VA, USA).

### 2.2. Cell Culture Conditions

The DMEM (Gibco; Ref 11320-033) was supplemented with 1% penicillin–streptomycin and 10% FBS solution and mixed gently before use. The Caco-2 cells were cultured as a monolayer in the prepared DMEM media in a cell culture T75 flask. Cells were allowed to adhere for 24 h in a humidified incubator set at 5% CO_2_ and 37 °C. Media were replaced every other day, allowing cells to grow to 90% confluency. Cells were then passaged, and 0.25% trypsin/EDTA was used to detach the cells before seeding, as required.

### 2.3. Particle Characterization

Silica particles were suspended at 0.1% *w*/*v* in 99:1 ultrapure water to dimethyl sulfoxide (DMSO) and bath sonicated for 50 min. Suspensions were diluted to a suitable attenuator setting and loaded into DTS 1070 folded capillary cells and measured using dynamic light scattering (DLS) and zeta potential (ZP) modes in a Zetasizer Nano ZS (Malvern Panalytical, Malvern, UK). Dry particles were also adhered to specimen mounts and sputter-coated using carbon thread. Mounted samples were imaged with 30kV accelerating voltage on an FEI Versa 3D scanning electron microscope (SEM, Thermo Fisher, Waltham, MA, USA). Particle dimensions were measured using ImageJ software (Rasband, 1997–2018). The length and width of 100 particles for each silica type were accurately measured and averaged.

### 2.4. Preparation of Silica Suspensions

Each silica morphology was prepared with DMEM media at six different concentrations using serial dilution. The concentrations prepared were 1000, 100, 10, 1, 0.1, and 0.01 ppm. Solutions were vortexed and then sonicated for 50 min to facilitate particle deagglomeration. Colloidal silica was sonicated for an extra 10 min for better suspension. All six concentrations were used to assess cellular response, while only 0.1 and 1000 ppm were used to quantify the ROS produced. DMSO (1%) was added to the solutions used to assess cellular response but excluded from those prepared for ROS quantification.

### 2.5. Cellular Response Assay

Cells (passages 4 to 6) were seeded in triplicate at a density of 2.0 × 10^4^ cells/well in a 96-well plate with 100 μL DMEM per well. Cells were incubated for 24 h to allow adherence. Cells were then exposed for 24 hr before assessing the mitochondrial activity using a tetrazolium compound 3-(4,5-dimethylthiazol-2-yl)-5-(3-carboxymethoxyphenyl)-2-(4-sulfophenyl)-2H-tetrazolium (MTS) kit (Cell Titer 96 AQueous One Solution Cell Proliferation Assay, Promega, Madison, WI, USA). As a positive control, 1% Triton X-100 (*v*/*v*) media were used. Post-exposure, cells were washed with PBS, and fresh media were added and supplemented with 20 μL of MTS dye. Cells were incubated for 2 h at 5% CO_2_ and 37 °C, and the absorbance was recorded at 490 nm to quantify the formazan dye using a microplate reader Biotek Synergy H1 Spectrometer (Agilent Technologies, Santa Clara, CA, USA). Mitochondrial activity after exposure was normalized to the negative control. Twelve replicates were averaged and reported with standard deviations as bar graphs.

### 2.6. Reactive Oxygen Species Production

Cells were seeded in triplicate at a 4.0 × 10^4^ cells/well density in a white 96-well plate with a transparent bottom and supplemented with 500 μL DMEM per well. Cells were incubated for 24 h to allow adherence. Cells were then exposed for 24 h. Menadione (50 μM) was diluted with DMEM media as a positive control. ROS-Glo^TM^ H_2_O_2_ assay (Promega, Madison, WI, USA) was then used to quantify the hydrogen peroxide produced. Luminescence was recorded on a BioTek Synergy H1 Spectrometer. The ROS levels were quantified as Relative Luminescence Units (RLUs). Twelve replicates were averaged and reported with standard deviations as bar graphs.

### 2.7. Statistical Analysis

Four experimental trials were performed in triplicate, and the results were normalized and averaged. Statistics were performed using GraphPad PRISM Version 10.2.1 (GraphPad Software, Inc., San Diego, CA, USA). Comparisons were made using the one-way ANOVA and the Tukey post hoc test to determine statistical significance. A *p*-value of <0.05 was defined as statistically significant unless stated otherwise.

## 3. Results

Particle characterization: The dynamic light scattering (DLS) and zeta potential (ZP) of aqueous suspensions of silica particles determined the average hydrodynamic diameter and charge for each type (Table 2). Pyrogenic silica had the smallest average size in suspension at 158 nm, while vitreous silica had the largest average size at 1534 nm. All silica suspensions had a negative charge in the stable regime.

**Figure 1:**
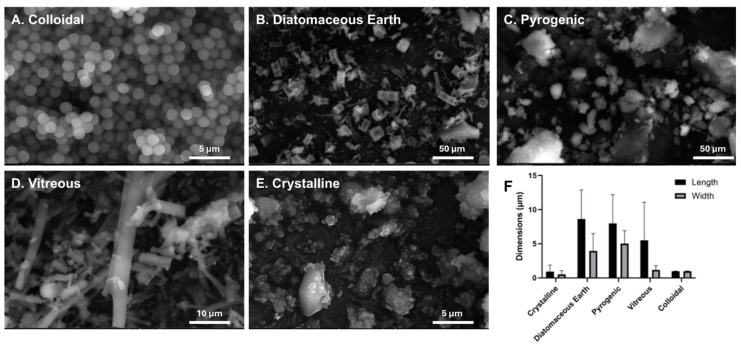
Scanning electron microscopy (SEM) images for each silica morphology at the indicated scale bars: (**A**) colloidal, (**B**) diatomaceous earth, (**C**) pyrogenic, (**D**) vitreous, and (**E**) crystalline. (**F**) Particle dimensions in micrometers, as determined by ImageJ analysis. The x-axis shows five distinct particles’ length and width parameters, providing an understanding of morphological diversity.

The scanning electron microscopy (SEM) of silica particles showed various sizes, morphologies, and surface characteristics (Figure 1). Particle dimensions measured using ImageJ revealed that diatomaceous earth and pyrogenic particles had the biggest dimensions of 8.649 × 3.956 µm and 7.971 × 5.031 µm, respectively. On the other hand, crystalline and colloidal particles were characterized by their smallest sizes of 0.923 × 0.521 µm and 0.96 × 0.96 µm, respectively.

**Figure 2:**
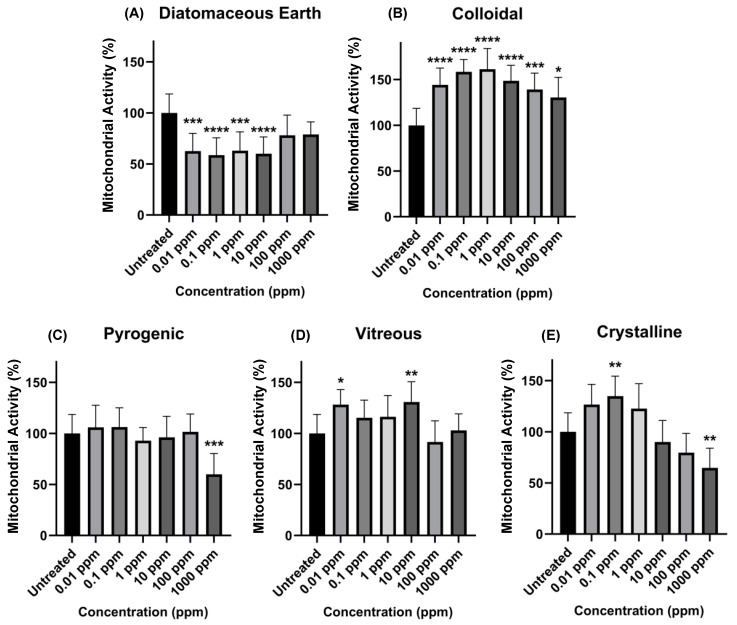
Percentage of mitochondrial activity calculated upon the exposure of Caco-2 cells to each silica type. Concentrations along the x-axis are reported in ppm, and the y-axis represents the percentage of mitochondrial activity after normalizing to the untreated. The asterisk (*) indicates a statistically significant difference between the untreated control and the exposed groups (* *p* ≤ 0.05, ** *p* ≤ 0.01, *** *p* ≤ 0.001, **** *p* ≤ 0.0001). Each panel represents the average and standard deviation across twelve data sets, where (**A**) represents diatomaceous earth, (**B**) represents colloidal, (**C**) represents pyrogenic, (**D**) represents vitreous, and (**E**) represents crystalline.

Cellular response: The differential cellular response, as measured by mitochondrial activity induced after inoculation exposure to the different silica morphologies in Caco-2 cells, was evaluated after a 24 h exposure period (Figure 2). Percent changes in mitochondrial activity relative to untreated cells were calculated. Exposure to 0.01, 0.1, 1, and 10 ppm diatomaceous earth decreased mitochondrial activity statistically significantly; however, at 100 and 1000 ppm exposure concentrations, no significant changes in cellular response were observed. Colloidal silica increased mitochondrial activity at all concentrations tested. Pyrogenic silica did not include changes in cellular response, except at the 1000 ppm exposure concentration, where mitochondrial activity decreased statistically significantly. Vitreous silica did not change cellular responses, except at the 0.01 and 10 ppm exposure concentrations, where mitochondrial activity increased statistically significantly. Similarly, crystalline silica did not change cellular responses, except at the 0.1 and 1000 ppm exposure concentrations, where mitochondrial activity increased statistically significantly.

The highest concentrations of 100 and 1000 ppm are generally regarded as overdose concentrations. Because silica readily aggregates, the cellular responses may not represent the same type of particle exposure as the exposure at 0.01 to 10 ppm. We tested the high particle concentrations to determine an EC50 value for each silica type. Results show that none of the silica morphologies induced a dose-response curve appropriate for reporting a half-maximal effective concentration. Nevertheless, we still conclude that a differential cellular response to microscale silica morphologies in human gastrointestinal cells is evident. In this study, colloidal silica induced the most significant increase in mitochondrial activity, followed by crystalline and vitreous; diatomaceous earth induced the most significant decrease in mitochondrial activity. Pyrogenic silica did not change the mitochondrial activity of cells.

**Figure 3:**
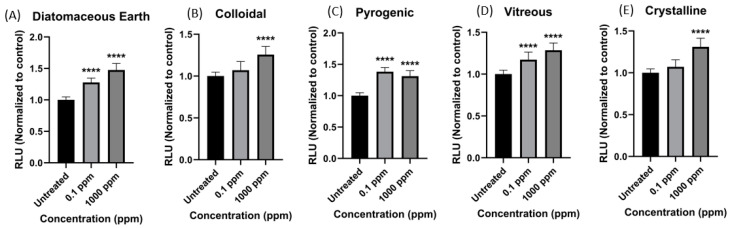
Bar graphs present the amount of hydrogen peroxide produced upon 24 h exposure of Caco02 cells to each silica type at two concentrations. (**A**) Diatomaceous earth; (**B**) colloidal; (**C**) pyrogenic; (**D**) vitreous; and (**E**) crystalline. The y-axis represents the relative luminescence units (RLUs) normalized to the untreated. Concentrations along the x-axis are reported in ppm. The asterisk (*) indicates a statistically significant difference between the control and the exposed groups (**** *p* ≤ 0.0001).

Reactive oxygen species generation: The production of H_2_O_2_ from Caco-2 cells after 24 h exposure to the five silica morphologies tested was assessed and normalized to the untreated cells as a measure of total reactive oxygen species (ROS) generation (Figure 3) and noted as RLUs (quantification of luminescence emitted by the chemical reaction). For this experiment, only two concentrations were tested, i.e., 0.1 and 1000 ppm. Compared to the untreated cells (where RLUs equal 1), diatomaceous earth and vitreous silicas prompted statistically significant H_2_O_2_ production at both concentrations and in a dose-dependent manner. Colloidal and crystalline silica induced statistically significant ROS production at the higher tested concentration (1000 ppm) with 1.26 ± 0.09 RLU and 1.32 ± 0.10 RLU production levels, respectively. At lower concentrations, the production of H_2_O_2_ was negligible. Pyrogenic silica prompted statistically significant H_2_O_2_ production at both concentration levels but not dose-dependently (at 1000 ppm, the RLU value was 1.32 ± 0.09 RLUs, and at 0.1 ppm, the RLU value was 1.39 ± 0.07 RLUs, (*p* < 0.0001)). The overall production of H_2_O_2_ among the five silica morphologies was insignificant compared to the 50 μM menadione positive control (RLU = 5.77).

Similar to the mitochondrial activity results, we conclude that a differential cellular response to microscale silica morphologies in human gastrointestinal cells is evident. In this study, diatomaceous earth induced the most increase in H_2_O_2_ production, followed by pyrogenic and vitreous; colloidal and crystalline induced the least amount of H_2_O_2_.

## 4. Discussion

Caco-2 cells, derived from human colorectal adenocarcinoma, are a valuable model for studying intestinal absorption [42,43]. This cell line, originally developed by Jorgen Fogh in 1975, has been widely validated for its relevance to intestinal absorption and cellular responses, making it a robust choice for this study [44]. Their resemblance to small intestinal epithelial cells’ structure and function makes them ideal for assessing how compounds are absorbed through the intestinal barrier [45]. They form polarized monolayers and express transport proteins and enzymes similar to those in the small intestine. This well-established characterization underscores their uses for studying drug absorption, metabolism, and bioavailability in vitro [46,47,48].

Zeta potential measures the electrostatic potential at the slipping plane between the particle surface and the surrounding liquid medium [49]. The negative zeta potential associated with the silica particles tested suggests that the particles have a net negative charge, and particles are likely to repel each other in solution, which can influence their interactions with other substances, including biological cells [50]. Like most mammalian cell membranes, the cell membrane of Caco-2 cells is typically electrically polarized, with a net negative charge on the inner side of the membrane and a net positive charge on the outer side [51,52]. This polarity arises from the distribution of charged molecules, such as phospholipids, proteins, and glycoproteins, across the cell membrane’s lipid bilayer [52]. The observed zeta potentials, falling from −12.7 mV to −29.8 mV, suggest a strong electrostatic repulsion between the particles and the Caco-2 cell membrane. This repulsion potentially inhibits the particles from interacting with and entering the cells, thereby minimizing cellular uptake and internalization processes. However, In some cases, particles with negative zeta potentials might still enter cells via endocytosis [53]. Silica microparticles with a negative charge and sharp edges can penetrate the negatively charged Caco-2 cell membrane through several mechanisms despite electrostatic repulsion. The sharp edges may physically disrupt the lipid bilayer, creating gaps or destabilizing the membrane. Additionally, interactions with membrane proteins or receptors can facilitate internalization via processes such as endocytosis [54,55,56]. Although surface modifications of the particles could potentially reduce repulsion or enhance compatibility with the cell membrane, in the absence of such modifications, other factors come into play. The DMEM media, which contain serum proteins, can adsorb onto the particles and act as a “bridge” that reduces direct electrostatic repulsion between the particles and the cell membrane, aiding in particle–cell interactions. Moreover, the ionic strength and pH of the media can influence the degree of repulsion or attraction, affecting how particles interact with the cell surface [57]. Furthermore, cellular uptake mechanisms, such as phagocytosis, can help overcome the repulsive forces and enable particles of sizes > 0.5 μM to cross the membrane [58].

The dispersity index (DI) is a dimensionless parameter used to characterize the width of the particle size distribution within a sample. A value closer to 0 indicates a more uniform distribution of particle sizes, while higher values suggest greater heterogeneity or variability in particle sizes [59]. In this context, the lower DI values for the colloidal (0.250) and pyrogenic (0.230) silica samples suggest relatively narrow size distributions, indicating a more uniform population of particles within these samples. On the other hand, the higher DI values for the vitreous (0.643), crystalline (0.490), and diatomaceous earth (0.470) silica samples indicate broader size distributions, implying greater variability in particle sizes within these samples.

The z-average particle size obtained from dynamic light scattering (DLS) measurements represents the mean hydrodynamic diameter of each particle. It provides crucial insights into their size distribution and aggregation state [60]. The highest z-average size of approximately 1534 nanometers, corresponding to the vitreous sample, indicates a significant proportion of larger particles and/or particle aggregates within the dispersion. Conversely, the smallest z-average size of around 158 nm, observed in the pyrogenic sample, signifies a relatively homogeneous population of smaller particles. The difference in particle size observed between ImageJ measurements and the z-average size can be attributed to the distinct methodologies and principles employed in these assessments. ImageJ provides a direct, static measurement of individual particle dimensions based on image analysis, reflecting the actual physical size observed [61]. Conversely, the z-average size, typically determined via dynamic light scattering (DLS), presents an intensity-weighted average size of suspended particles, incorporating the hydrodynamic radius, encompassing the particle and its surrounding solvent layer [62]. This averaging process can yield different results, especially in polydispersed samples, leading to variations between the two measurements.

Our analysis, including ImageJ measurements from SEM images, confirmed that the silica materials contained primarily micrometer-sized particles, consistent with the vendor’s specifications. DLS analysis further verified the absence of significant nanometer-sized components. This suggests that our results reflect micrometer-sized silica interactions with Caco-2 cells without the confounding effects of nanometer-sized particles. However, the potential impact of nanometer-sized components in other silica materials remains crucial for the field, needing further investigation.

The MTS assay evaluates cell viability by measuring mitochondrial activity, as active mitochondria in viable cells reduce MTS to a colorimetric formazan product. Higher mitochondrial activity correlates with greater cell viability, reflecting the overall health and function of the cells [63]. The lack of mitochondrial activity changes after exposure to pyrogenic silica implies no cytotoxicity to the cells under the tested conditions. On the other hand, diatomaceous earth displayed the lowest mitochondrial activity among all five types of silica particles tested. The decline in the cellular mitochondrial activity exposed to diatomaceous earth (i.e., 62% at 0.01 ppm to 78% at 1000 ppm) is attributed to several factors. Given its large dispersity index and hydrodynamic diameter coupled with its low zeta potential value, diatomaceous earth silica particles are prone to aggregation, potentially affecting particle-to-particle and particle-to-cell interactions. Also, diatomaceous earth has more impurities than any of the other silica types tested. Crystalline and colloidal silica yielded insights into the material’s impact on cell viability, revealing a concentration-independent response characterized by increased mitochondrial activity at lower concentrations and a statistically significant decrease at higher concentrations [64]. This aligns well with previous studies [65].

Reactive oxygen species (ROS) are a group of highly reactive molecules containing oxygen, such as hydrogen peroxide, superoxide, hydroxyl radical, and singlet oxygen [66,67]. The primary origin of ROS production is mitochondria, which are released due to cellular metabolism [68,69,70]. The induction of low levels of H_2_O_2_ upon exposure to the silica types tested may signify cellular defense mechanisms or adaptive responses by the Caco-2 cells. Like other cell types, Caco-2 cells develop antioxidant defense mechanisms to mitigate ROS-induced damage, which could effectively neutralize H_2_O_2_ and limit its accumulation within the cells [71].

Following oral uptake, silica microparticles are generally poorly absorbed and primarily remain in the gastrointestinal tract, excreted through feces within a few hours [19,72]. During a 24 h exposure, the degradation of these particles is minimal, as they are typically resistant to rapid breakdown and persist largely intact through the digestive system [73]. The exact time to excretion and the degree of degradation can vary based on particle size and surface properties.

A 24 h exposure study, while providing critical initial data on the immediate effects of silica particles, has notable limitations compared to chronic exposure studies. Short-term exposure primarily reveals acute responses and may not fully capture the long-term effects, such as cumulative toxicity, chronic inflammation, or progressive cellular damage that can develop over extended periods. This brief exposure period might overlook the body’s adaptive responses and fail to account for potential long-term health outcomes, including carcinogenicity or organ damage. Chronic exposure studies are essential for a comprehensive understanding of how prolonged or repeated exposure impacts biological systems upon particle degradation, offering a more detailed assessment of safety and health risks. Thus, while a 24 h study is useful for evaluating immediate effects, it is important to conduct long-term studies to assess particle degradation and potential chronic toxicity.

The endpoints collected in this study include mitochondrial activity, ROS production, dispersity index, hydrodynamic diameter, and zeta potential. The results revealed four specific relationships. First, mitochondrial activity was inversely proportional to ROS production, with the silica morphologies ranked in this order: diatomaceous earth, pyrogenic, vitreous, colloidal, and crystalline. Second, the dispersity index was proportional to hydrodynamic diameter, with the order rank of pyrogenic, colloidal, crystalline, diatomaceous earth, and vitreous. Third, the mitochondrial activity was proportional to the zeta potential, with the order rank of colloidal, crystalline, vitreous, pyrogenic, and diatomaceous earth. Fourth, the ROS generation is inversely proportional to the zeta potential, with the order rank of diatomaceous earth, pyrogenic, vitreous, colloidal, and crystalline.

The results of this study provide insights into the cellular responses of Caco-2 cells to silica micrometer-sized particles. However, the study’s design limits its applicability to more complex gastrointestinal scenarios. The absence of a mucus layer in the Caco-2 model means that the interactions observed may not fully represent in vivo conditions, where this layer is important in protecting intestinal epithelium and altering particle interactions [74,75]. Additionally, the gut microbiota’s influence on the toxicity of silica is a critical factor that needs further investigation. Future research is needed.

## 5. Conclusions

Our findings demonstrate that diatomaceous earth decreased mitochondrial activity in Caco-2 cells, while colloidal silica increased the activity. Cellular hydrogen peroxide production increased after exposure to all five silica types. These results indicate that the impact of silica types on Caco-2 cell health varies, with some showing dose-dependent responses and others exhibiting more complex patterns. Evidence shows correlations among mitochondrial activity, ROS production, and zeta potential. The dispersity index is correlated to the hydrodynamic diameter for these silica types. More studies that investigate the differential cellular responses to the same material but with differing morphologies need to be performed, and these responses are probably due to variations in particle physicochemical properties and compensating cellular responses. While mechanistic analyses are needed to elucidate the type of toxicity induced, the results suggest that workers exposed to these silica particles could be at risk of adverse health effects. Further research is needed to fully understand the long-term implications and to develop appropriate safety measures.

## Figures and Tables

**Table 1:** The specifics of the silica purchased for conducting the study, as provided by each manufacturer.

Type	Vendor	Name	CAS	Origin	Country
Crystalline	Honeywell	Quartz (SiO_2_) 99%, 1–5 μm	14808-60-7	Germany	Muskegon, MI, USA
Colloidal	Thermo Fisher Scientific	Silicon (IV) oxide, powder, 1.0 micron, 99.9%	7631-86-9	USA	Waltham, MA, USA
Vitreous	Sigma-Aldrich	Vitreous milled nanofiber, 90–100%	60676-86-0	Czech Republic	St. Louis, MO, USA
Diatomaceous Earth	Harris	Ground freshwater diatomaceous earth (fossil shell flour), 100%	61790-53-2	USA	Cartersville, GA, USA
Pyrogenic	Thermo Fisher Scientific	Silicon (IV) oxide, amorphous fumed, 325-mesh powder, S.A. 350–420 m^2^/g	7631-86-9	Germany	Waltham, MA, USA

**Table 2:** Summary of the dispersity index, hydrodynamic diameter, and zeta potential of each silica.

Type of Silica	Dispersity Index	Hydrodynamic Diameter (nm)	Zeta Potential (mV)
Colloidal	0.250	1015 ± 31	−29.8 ± 1.0
Pyrogenic	0.230	158 ± 0.6	−16.1 ± 1.5
Vitreous	0.643	1534 ± 115	−17.6 ± 0.8
Crystalline	0.490	1068 ± 41	−19.3 ± 0.5
Diatomaceous Earth	0.470	1263 ± 92	−12.7 ± 0.6

## Data Availability

Data are available upon request.
